# Targeting Androgen Receptor in Treating HER2 Positive Breast Cancer

**DOI:** 10.1038/s41598-017-14607-2

**Published:** 2017-11-06

**Authors:** Licai He, Zhuanyun Du, Xusheng Xiong, Hua Ma, Zhenfeng Zhu, Hongwei Gao, Jiawei Cao, Tong Li, Hongzhi Li, Kaiyan Yang, Guorong Chen, Jennifer K. Richer, Haihua Gu

**Affiliations:** 1 Key Laboratory of Laboratory Medicine, Ministry of Education, School of Laboratory Medicine and Life Science, Wenzhou Medical University, Wenzhou, 325035 China; 2 Department of Pathology, The First Affiliated Hospital of Wenzhou Medical University, Wenzhou Medical University, Wenzhou, 325035 China; 3 Department of Pathology, University of Colorado Anschutz Medical Campus, Aurora, Colorado, 80045 USA

## Abstract

Androgen receptor (AR) is widely expressed in different subtypes of breast cancer (BC). However, it is unclear how AR functions in HER2 positive (+) BC. Knockdown of AR with shRNAs and a new generation anti-androgen drug, Enzalutamide, were used to explore the involvement of AR on the growth of HER2 + BC cells (HCC1954 and SKBR3). AR shRNA or Enzalutamide inhibited the growth of SKBR3 cells at a similar extend compared to trastuzumab, an approved HER2 targeted drug. Combining Enzalutamide with trastuzumab further decreased the growth of HCC1954 and SKBR3 cells compared with single agent alone *in vitro*. Biochemical analysis revealed that inhibiting AR resulted in decreased HER2 phosphorylation and activation of Erk and Akt, without affecting the HER2 and HER3 expression. The *in vivo* efficacy of Enzalutamide was further tested using the HCC1954 xenograft model. Enzalutamide impaired the growth of HCC1954 tumor at a level comparable to that by trastuzumab. Enzalutamide decreased Ki67 staining and increased activated caspase3 staining compared with vehicle control in HCC1954 tumors. Our results indicate AR plays an important role in promoting the growth of HER2 + BC by cross-talking with the HER2 signaling. AR drug may be used as an alternative second line therapy for treating HER2 + BC.

## Introduction

HER2 positive (+) breast cancer accounts for ~25% of breast cancer and has a poor prognosis^1^. Clinically, HER2 + breast cancer is identified by immunochemistry (IHC) of HER2 with a 3 + staining or fluorescent *in situ* hybridization (FISH) for *HER2* ^2^. HER2 mainly via heterodimerization with HER3^3^ activates downstream signaling pathways including PI3K/Akt and Ras/Erk to drive the growth of the breast cancer cells^4,5^. Various HER2 targeted therapies have been developed over the years^6,7^. Trastuzumab (Herceptin), a humanized monoclonal antibody against HER2, when combined with chemotherapy, is very effective in treating HER2 + breast cancer, with a 50–80% response rate^8^. However, 20–50% of patients with HER2 + tumors do not respond to trastuzumab initially or develop acquired resistance after one year of treatment^9^. Thus, it is important to explore alternative second line therapies.

Androgen receptor (AR) is a ligand-activated transcription factor that belongs to the steroid hormone receptor family. AR signaling is well known to drive the growth of prostate cancer cells as reviewed^10^ and recent studies reveal that AR is more widely expressed than estrogen receptor (ER) or progesterone receptor (PR) in breast cancer^11,12^. According to the Nurses’ Health Study in 2011, AR expression can be detected in 77% of the breast cancer with the breakdown of 88% of ER+, 59% of HER2+, and 32% of triple negative breast (TNBC) expressing AR respectively^12^. Although as with ER, AR expression in breast has been largely associated with a better prognosis than AR negative disease,^13–16^, recent studies indicate that AR promotes the growth of ER + breast cancer^17^ and TNBC^18,19^ via distinct mechanisms. In ER + BC, studies from the use of a new generation of anti-androgen drug, Enzalutamide (also known as MDV3100)^20^, indicate that AR signaling is required for androgen and estrogen-induced tumor cell growth *in vitro* and *in vivo* ^17^. In the luminal AR^18,21^ subset of TNBC, AR activity is required for the growth of LAR-TNBC through the upregulation of HER3^19^. In other subset of TNBC, AR activity promotes the growth of TNBC at least in part via upregulating the expression of amphiregulin^22^. Various clinical trials using androgen antagonists bicalutamide and Enzalutamide in treating AR + TNBC are ongoing (clinicaltrials.gov: NCT01889238, and NCT00468715).

In contrast, the role of AR in HER2 + breast cancer is less well understood. The prognostic value of AR expression have been reported to be associated with either better^23^ or worse^14^ patient survival of HER2 + breast cancer (BC). Previous studies suggested a role of AR in the molecular apocrine BC (maBC), which is a subtype of estrogen receptor negative (ER-) BC that overexpresses AR and frequently HER2 as well^24^. Treating MDA-MB-453 cells, a maBC line, with the first generation anti-androgen drug flutamide reduced cell proliferation and induced apoptosis of maBC *in vitro* ^25^. Furthermore, AR promoted Erk activation via upregulation of HER2 gene transcription^26^. Considering the role of AR and its interaction with HER2 in maBC, we hypothesize that AR expression may contribute to the growth of HER2 + BC.

In the current study, we examined the functional role of AR in HER2 + BC by blocking AR expression with shRNA and AR activity with androgen antagonist Enzalutamide in HER2 + BC cell lines, SKBR3 and HCC1954. These two cell lines are well known models of HER2 + BC that are estrogen receptor negative. Considering the reported interaction between AR and estrogen receptor in breast cancer^27^, our study should help dissect the role of AR in HER2 + BC independent of ER. Our data revealed that inhibiting AR impairs the growth of HER2 + breast cancer cells *in vitro* and vivo, in comparable to the effect of trastuzumab. AR inhibition also reduced HER2 phosphorylation and activation of Akt and Erk without affecting HER2 and HER3 protein expression. Our results suggest that AR plays a novel role in HER2 signaling and AR targeting therapy may be useful in treating HER2 + breast cancer.

## Results

### Inhibiting AR impairs the growth of HER2 + breast cancer cells

**Figure 1:**
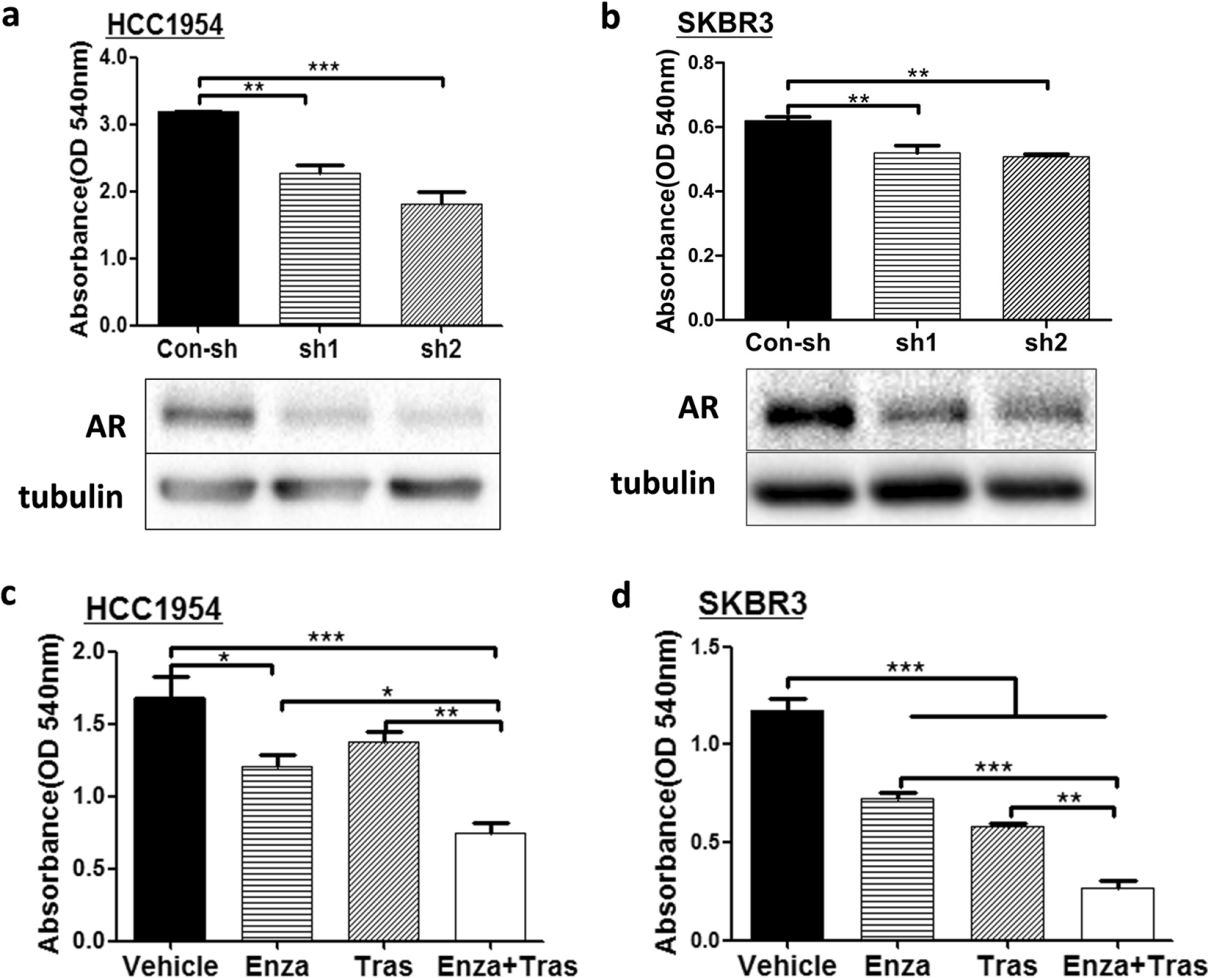
Inhibition of AR impairs the growth of HER2 breast cancer cells. (**a**,**b**) Knockdown of AR with shRNAs reduced the growth of HCC1954 and SKBR3. HCC1954 (**a**) and SKBR3 (**b**) cells were infected with lentivirues expressing control shRNA (Con-sh) and two different AR shRNAs (sh1 and sh2) and immunoblotted with anti-AR and anti-tubulin antibodies (**a**,**b** bottom panels). All cropped blots were run under the same experimental conditions. The original blots are included in Supplementary Figure S2. Colony formation assay was used to assess the growth of HCC1954 and SKBR3 cells expressing Con-sh, sh1, and sh2 (**a**,**b** top panels). AR antagonist, enzalutamide (Enza), together with trastuzumab (Tras) inhibited the growth of HCC1954 (**c**) and SKBR3 (**d**) cells. Cells were subjected to colony formation assay in the presence of Vehicle, 20 μM Enza, 20 μg/ml Tras, and 20 μM Enza + 20 μg/ml Tras for 6 days before analyzed with crystal violet staining. Data shown is the representative from three independent experiments. *p < 0.05, **p < 0.01, and ***p < 0.001, one-way ANOVA.

To test whether AR can drive the growth of HER2 + breast cancer, AR shRNAs were used to knockdown AR expression in HCC1954 and SKBR3. Both of the AR shRNAs significantly inhibited AR protein expression in HCC1954 and SKBR3 cells as indicated from the western blot analysis (Fig. 1a and b bottom panels). Furthermore, data from colony forming assays revealed that AR shRNA impaired the growth of HCC1954 (Fig. 1a, top panel) and SKBR3 (Fig. 1b, top panel) cells compared to control shRNA. Similar results were obtained when the growth of HCC1954 and SKBR3 was analyzed using CCK-8 reagent (Supplementary Fig. S1a,b), further supporting that knockdown of AR expression reduced the growth of HER2 + breast cancer cells.

To further test whether AR is a potential target for treatment of HER2 + breast cancer, Enzalutamide (Enza), an FDA approved AR targeting drug, was used to treat HCC1954 and SKBR3 cells *in vitro* using colony formation assay. In addition, the effects of trastuzumab alone or together with Enza on the growth of HCC1954 and SKBR3 cells were also assessed (Fig. 1c and d). Similarly to AR knockdown, Enza treatment alone resulted in significant inhibition of cell growth in both cell lines. In SKBR3 cells, the effect of Enza treatment was similar to the inhibitory effect of trastuzumab (Fig. 1d). Although trastuzumab alone had minimal effect on the growth of HCC1954 cell (Fig. 1c), treatment with both Enza and trastuzumab led to further reduction in cell growth compared with treatment with either single agent in both cell lines (Fig. 1c and d). Again, similar observations were seen when the effects of Enza and Trastuzumab on the growth of HCC1954 and SKBR3 were analyzed using CCK-8 reagent (Supplementary Fig. S1c,1d). These data indicate that AR plays a critical role in the growth of HER2 + breast cancer cells.

### AR is important for HER2 activation and signaling, independent of HER3 protein expression, in HER2 + breast cancer cells

**Figure 2:**
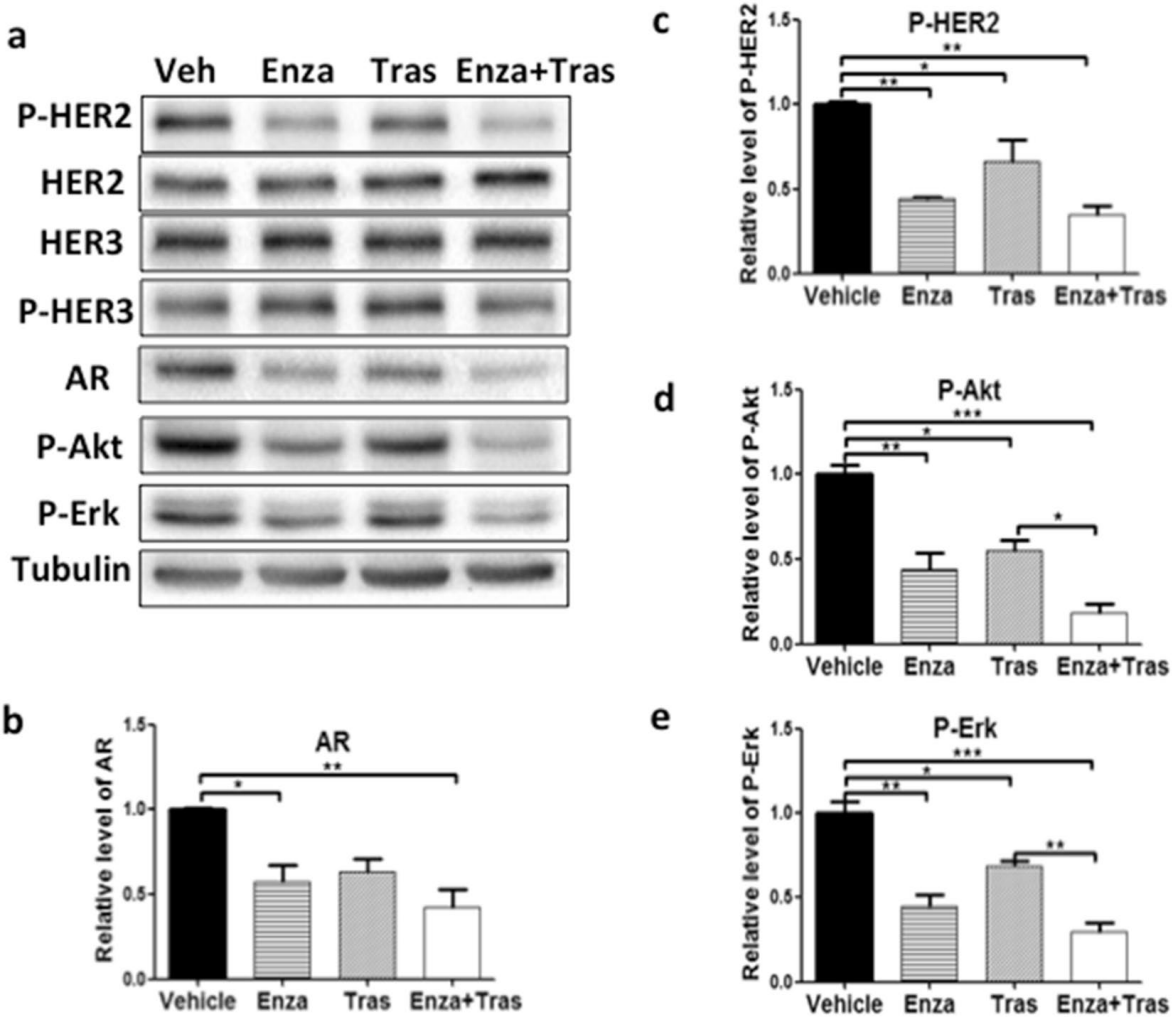
Enzalutamide inhibits HER2 phosphorylation and signaling without affecting HER3 protein expression in SKBR3 cells. Equal amounts of lysates from SKBR3 cells treated with Vehicle, 20 μM Enza, 20 μg/ml Tras, and 20 μM Enza + 20 μg/ml Tras for 4 days were immunoblotted with the indicated antibodies (**a**). All cropped blots were run under the same experimental conditions. The original blots are included in Supplementary Figure S3. The relative levels of AR (**b**), P-HER2 (**c**), P-Akt (**d**), and P-Erk (**e**) were quantified by normalizing with the Tubulin levels. Data shown is the average from three independent experiments. *p < 0.05, **p < 0.01, and ***p < 0.001, one-way ANOVA.

**Figure 3:**
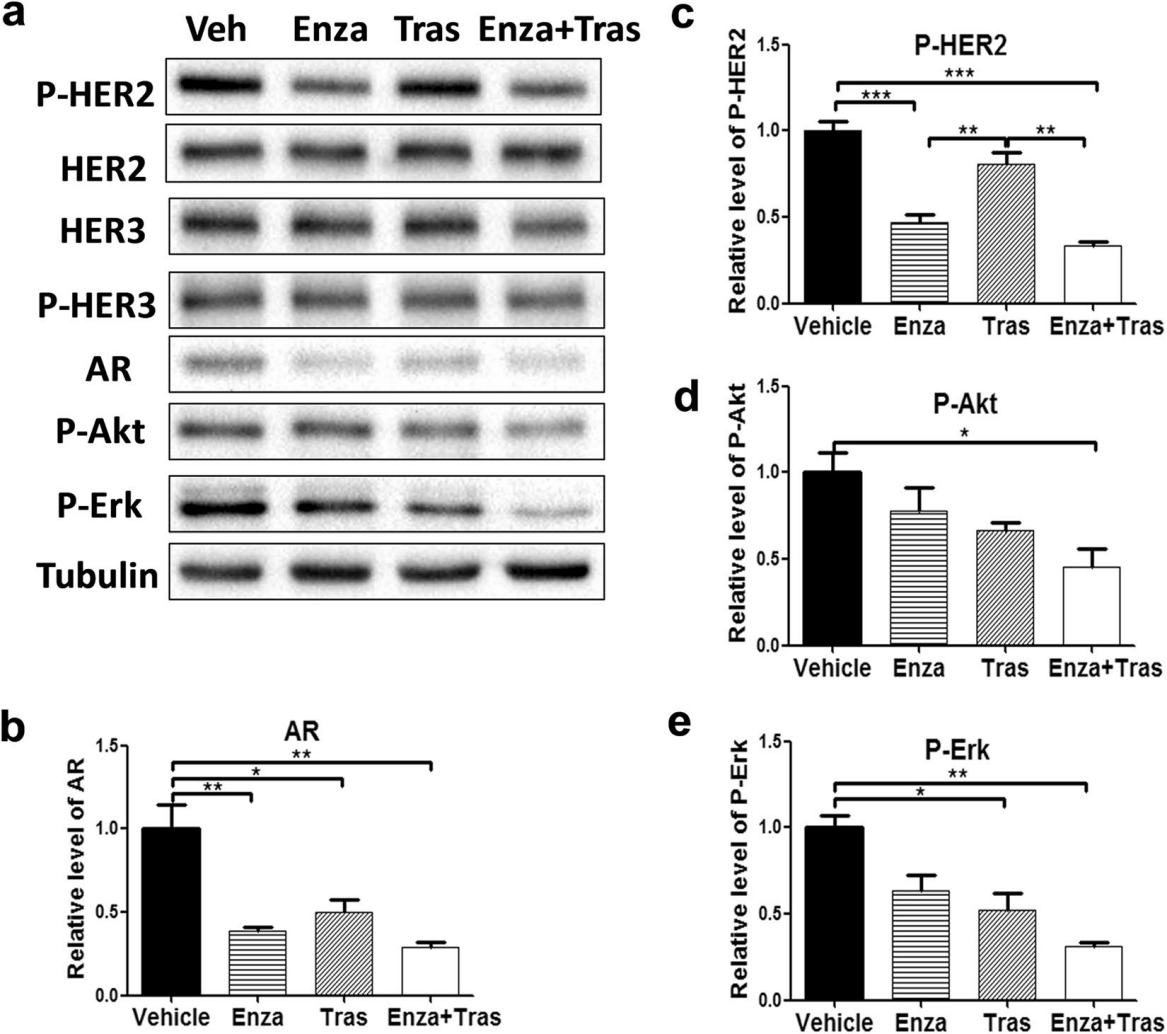
Enzalutamide inhibits HER2 phosphorylation and signaling without affecting HER3 protein expression in HCC1954 cells. Equal amounts of lysates from HCC1954 cells treated with Vehicle, 20 μM Enza, 20 μg/ml Tras, and 20 μM Enza + 20 μg/ml Tras for 6 days were immunoblotted with the indicated antibodies (**a**). All cropped blots were run under the same experimental conditions. The original blots are included in Supplementary Figure S4. The relative levels of AR (**b**), P-HER2 (**c**), P-Akt (**d**), and P-Erk (**e**) were quantified by normalizing with the Tubulin levels. Data shown is the average from three independent experiments. *p < 0.05, **p < 0.01, and ***p < 0.001, one-way ANOVA.

**Figure 4:**
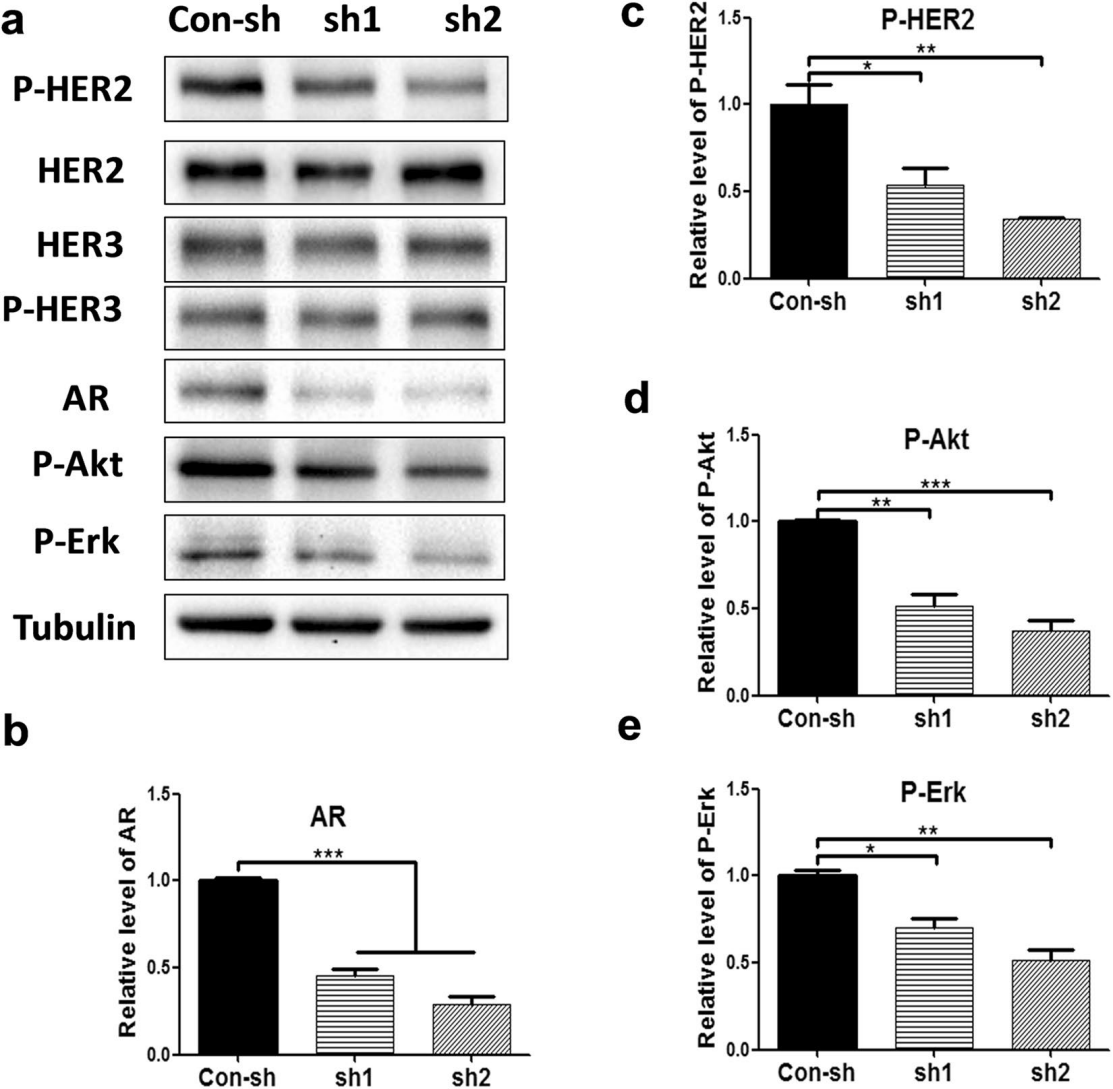
Knockdown of AR expression inhibits HER2 phosphorylation and signaling without affecting HER3 protein expression in HCC1954 cells. Equal amounts of lysates from HCC1954 cells expressing con-sh, AR sh1, and AR sh2 were immunoblotted with the indicated antibodies (**a**). All cropped blots were run under the same experimental conditions. The original blots are included in Supplementary Figure S5. The relative levels of AR (**b**), P-HER2 (**c**), P-Akt (**d**), and P-Erk (**e**) were quantified by normalizing with the Tubulin levels. Data shown is the average from three independent experiments. *p < 0.05, **p < 0.01, and ***p < 0.001, one-way ANOVA.

Because a previous report showed that AR activates HER2 signaling via upregulation of HER3 expression in luminal AR triple negative cells lines (MB-453 and SUM185PE)^18^. Western blot analysis was used to examine the phosphorylation status of HER2, HER3, and total HER2, HER3 protein expression in HER2 + breast cancer cells treated with Enza (Fig. 2). Compared with vehicle control, Enza treatment, which reduced AR protein expression, significantly decreased HER2 phosphorylation in HCC1954 and SKBR3 cells (Figs 2a,c and 3a,c). In addition, Enza treatment also reduced activation of Akt and Erk (Figs 2d,e and 3d,e), known downstream pathways of HER2. However, Enza treatment did not inhibit HER3 and HER2 total protein expression (Figs 2a and 3a). Likewise, knockdown of AR expression by shRNA impaired HER2 phosphorylation, Akt and Erk activation without affecting HER3 and HER2 protein expression in HCC1954 (Fig. 4). Interestingly, trastuzumab treatment inhibited HER2 phosphorylation as well as Akt and Erk activation in the similar extent as Enza treatment in SKBR3 cells (Fig. 2a). These data indicate that AR plays an important role in HER2 activation and signaling, without affecting HER2 and HER3 protein level.

### Enzalutamide is effective in inhibiting HER2 + breast cancer in xenograft model

Because of its efficacy in reducing the growth of HER2 + breast cancer cells *in vitro*, the effect of Enza on HER2 + tumor growth was examined in HCC1954 xenograft model. HCC1954 cells mixed with matrigel were injected into both flanks of the Balbc/nude mice. Mice were monitored for tumor growth by palpation and caliper measurement, and randomized into four groups for treatment with Vehicle, Enza, Trastuzumab, or Enza + Trastuzumab two weeks after cell injection.

**Figure 5:**
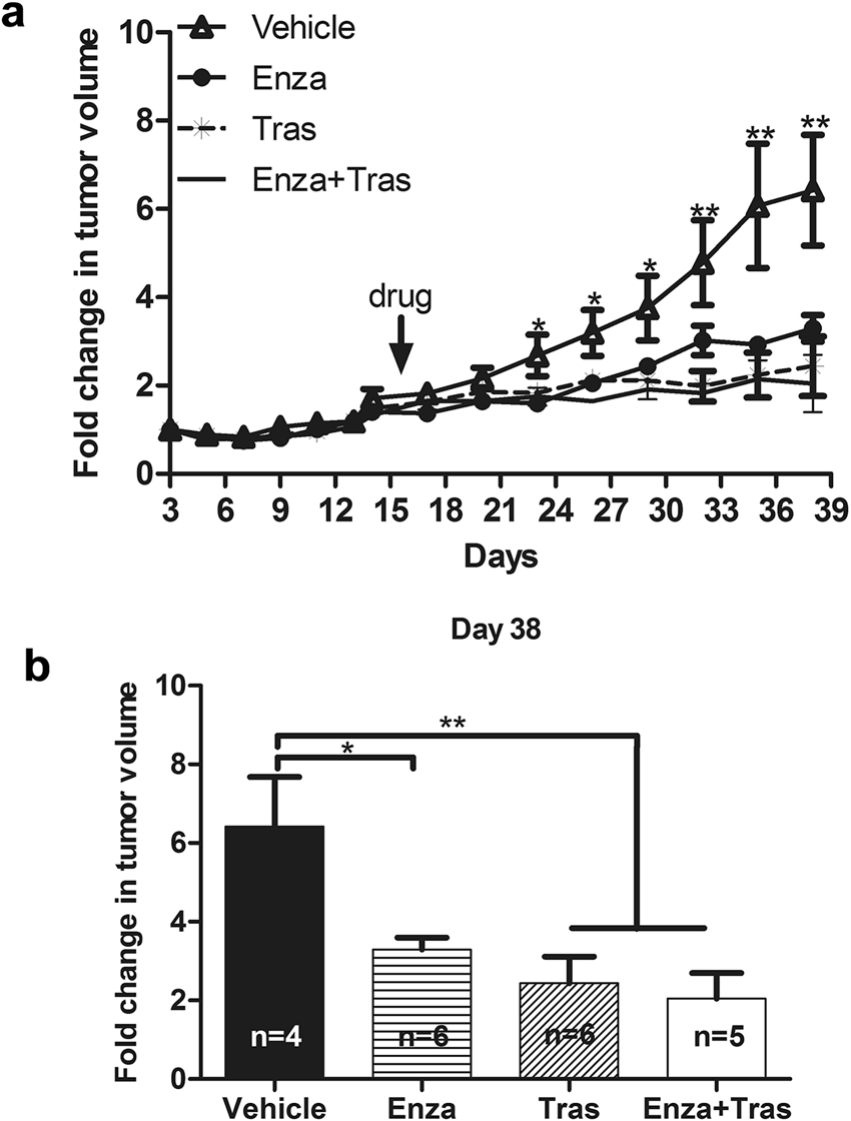
Enzalutamide inhibits HER2 + tumor growth in HCC1954 xenograft model. HCC 1954 cells were injected into the flanks of Balbc/nude mice. Sixteen days later, mice were randomized and treated with Vehicle, Enza, Tras, and Enza + Tras for 21 days. Tumor growth was monitored by caliper measurement every 3 days (**a**). (**b**) Relative tumor growth on day 36 after initial HCC1954 cell injection into the mice. Relative tumor growth was shown as fold change in tumor volume compared with the tumor size on day 3 after initial cell injection. n = the numbers of mice used in each treatment group. *p < 0.05, and **p < 0.01, one-way Anova. Data shown is representative from two independent experiments.

While HCC1954 tumors treated with vehicle control grew gradually, Enza treatment significantly decreased the tumor growth. Likewise Trastuzumab treatment dramatically reduced HCC1954 tumor growth (Fig. 5a). Addition of Enza to Trastuzumab did not significantly further reduce the growth of HCC1954 tumors compared with Trastuzumab treatment alone. This indicates that Enza efficacy is similar to that of Trastuzumab (Tras) in preclinical model of HER2 breast cancer (Fig. 5a, and b).

**Figure 6:**
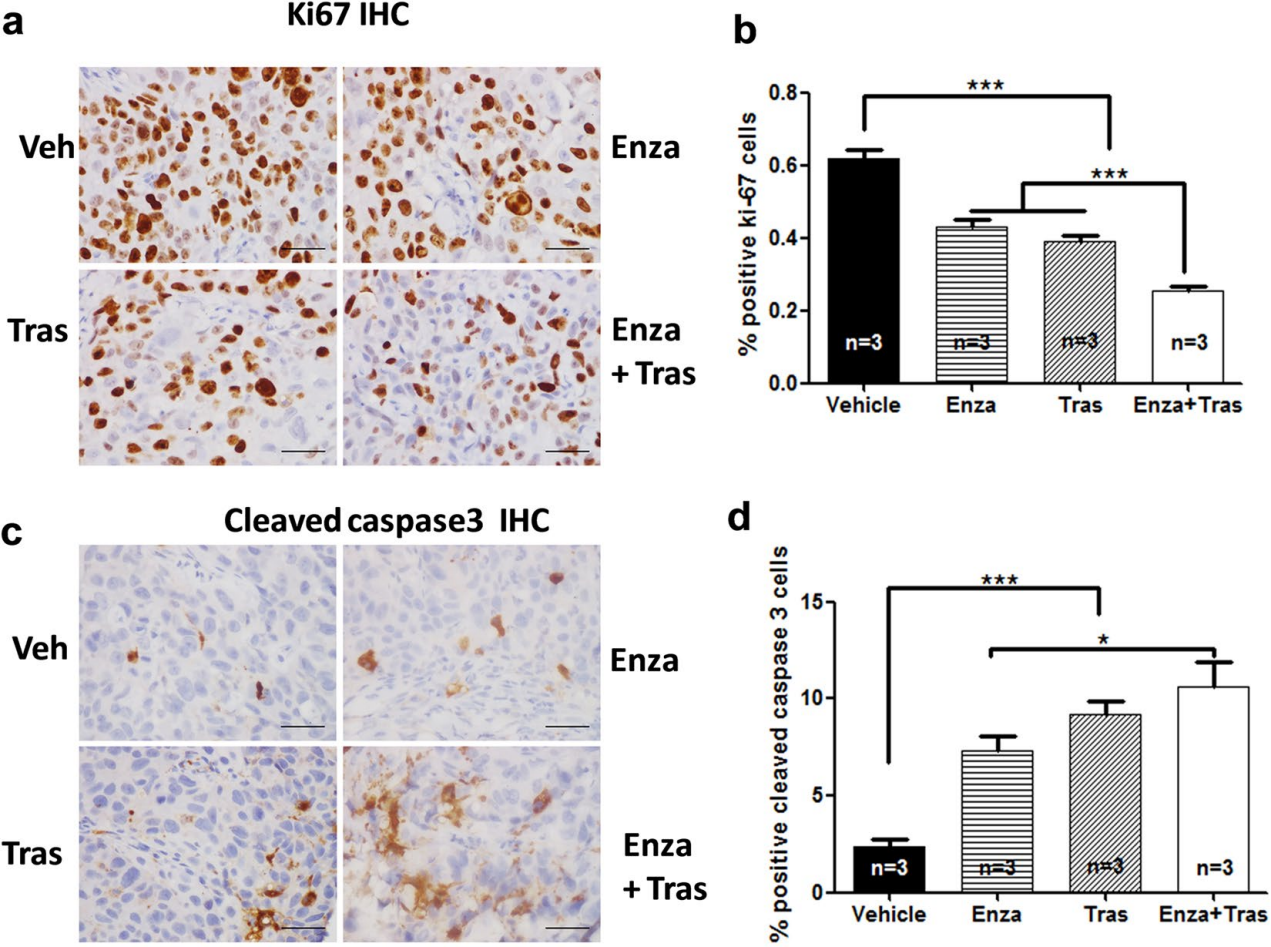
Enzalutamide inhibits cell proliferation and increases cell death in HER2 + xenograft tumors. Tumors from mice treated with Vehicle, Enza, Tras, Enza + Tras for 21 days were subjected to IHC staining with Ki67 antibody (**a**,**b**) and cleaved Casp3 antibody (**c**,**d**). n = 3 mice for each group. Scale bar = 50 μm. *p < 0.05, and ***p < 0.001, one-way ANOVA.

Immunohistochemistry (IHC) using antibodies against Ki67 and activated caspase 3 (Casp3) was used to examine the effects of Enza on cell proliferation and apoptosis respectively in xenograft tumors (Fig. 6). Compared with vehicle control, Enza and Tras treatments decreased Ki67 staining by ~33% (p < 0.001) and ~35% (p < 0.001) respectively whereas Enza plus Tras treatment reduced Ki67 staining by ~50% (Fig. 6a and b). While vehicle control tumors displayed ~2.5% Casp3 stained (+) cells, tumors treated with Enza and Tras showed ~7% (p < 0.001) and 9% (p < 0.001) Casp3 + cells. Addition of Enza to Tras further increased Casp3 + cells compared with Enza alone (p < 0.05) (Fig. 6c and d).These data revealed that Enza inhibits HCC1954 tumor growth via reduced cell proliferation and increased cell death, similar to the effects of trastuzumab on HCC1954.

**Figure 7:**
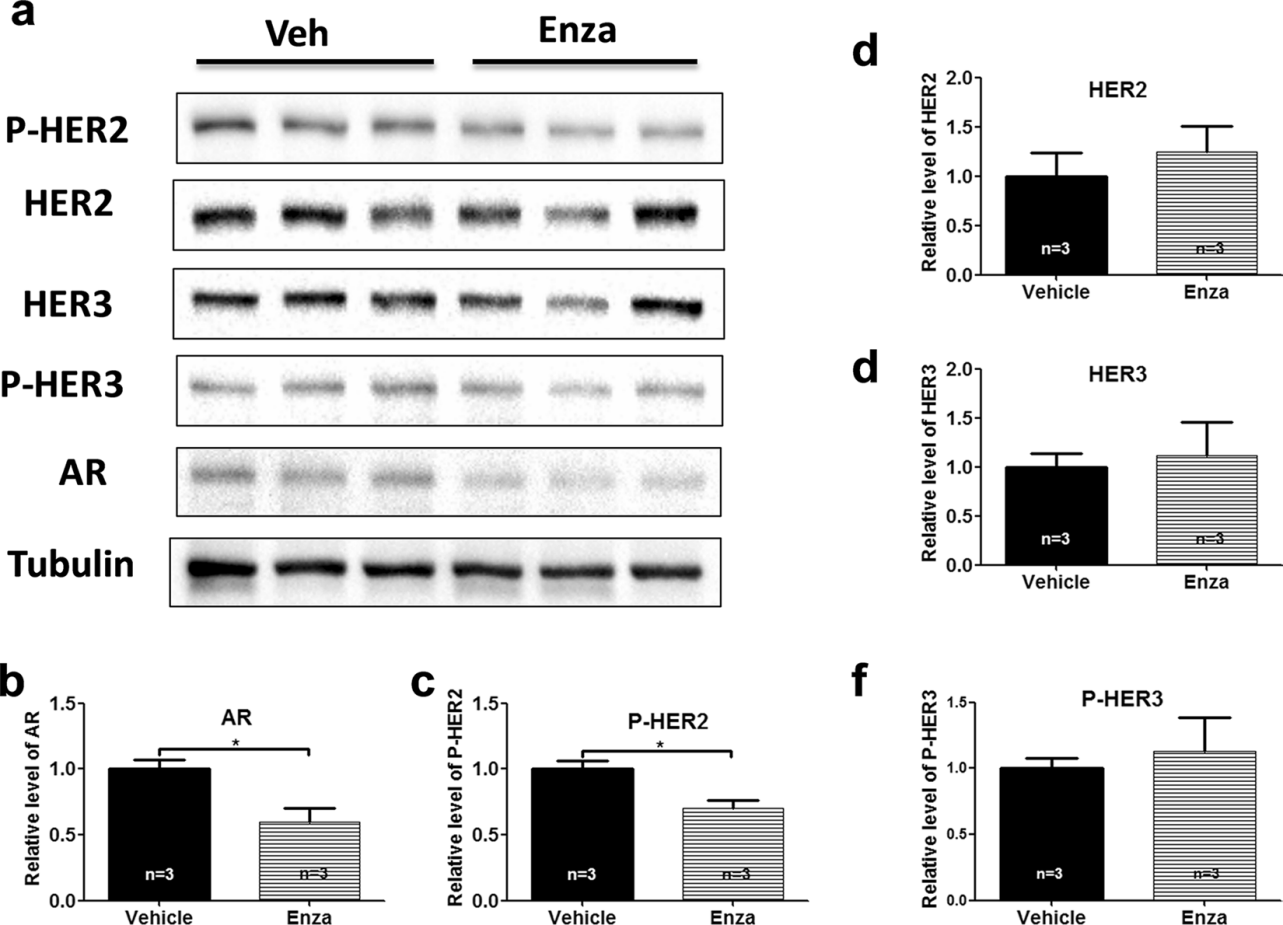
Enzalutamide inhibits HER2 phosphorylation and signaling without affecting HER3 protein expression in HER2 + xenograft tumors. Tumor lysates from mice (three mice each group) treated with Vehicle and Enza for 21 days were immunoblotted with the indicated antibodies (**a**). All cropped blots were run under the same experimental conditions. The original blots are included in Supplementary Figure S6. The relative levels of AR (**b**), P-HER2 (**c**), HER2 (**d**), HER3 (**e**), and P-HER3 (**f**) were quantified by normalizing with the Tubulin levels. *p < 0.05, two-tailed student’s t-test.

Western blot analysis was used to examine the effect of Enza on HER2 signaling in xenograft tumors. Similarly to what was found *in vitro* (Fig. 3), Enza treatment reduced AR expression, and inhibited HER2 phosphorylation without affecting HER2 and HER3 protein expression compared with vehicle treatment in HCC1954 xenografts (Fig. 7).

## Discussion

AR is widely expressed in HER2 + breast cancer; however, its function in HER2 + breast cancer is unclear. Results from this study suggest AR can in part activate HER2 signaling to drive the growth of HER2 + breast cancer cells *in vitro* and *in vivo*. Targeting AR can be used as an alternative therapy to treat HER2 + breast cancer.

Our data suggest that AR acts in a distinctive mechanism to promote the growth of HER2 + breast cancer cells. Our data reveal that AR contributes to HER2 phosphorylation and the activation of its downstream signaling pathway including Akt and Erk. In addition, AR inhibition did not affect HER2 and HER3 protein expression in HER2 + breast cancer cells (Figs 2, 3, 4, and 7). This is different from the action of AR in LAR-TNBC (i.e. MDA-MB-453 and SUM185PE) where AR enhances HER2 phosphorylation and signaling by increasing the expression of HER3 protein^19^. Specially, AR signaling promoted the transcription of WNT7B, which activates Wnt signaling. Then, AR complexed with the Wnt-activated beta-catenin upregulated transcription of *HER3* ^19^ in LAR-TNBC cells. In addition, AR signaling had been shown to enhance the transcription of *HER2* gene in maBC^26^. In contrast, our results support a model that AR activation may be important for maximal HER2 activation without affecting the expression of *HER2* and *HER3* in a subset of HER2 + breast cancer.

There are several mechanisms by which AR may activate HER2 in HER2 + breast cancer cells. One is that AR signaling may increase the expression of a ligand for HER3, which is a heterodimeric partner for HER2. Consistent with such a hypothesis, AR can contribute to the expression of amphiregulin, a ligand for EGFR, which is highly expressed in some TNBC^22^. It is also possible that AR signaling may inhibit the expression and activities of specific protein tyrosine phosphatases that can dephosphorylate and inactivate HER2^28,29^. Future studies are certainly required to uncover the detail mechanism of AR action in HER2 + breast cancer.

Two well-known HER2 + breast cancer cell lines, SKBR3 and HCC1954, were used in our study. Our data showed that SKBR3 cells were sensitive (Fig. 1d) whereas HCC1954 cells were resistant (Fig. 1c) to trastuzumab treatment *in vitro*, which are in agreement with published studies by other group^30,31^. However, trastuzumab was quite effective in inhibiting the growth of HCC1954 tumor in our xenograft model (Fig. 5a), which is not consistent with other published studies. The unique xenograft model, HCC1954 injected into the flank of BALB/c-*nu/nu* mice, used in our study might be account for this discrepancy. In studies with reported trastuzumab resistance by the HCC1954 models, different strains of immune deficient host mice were used, which include *nu/nu: Alpk* ^32^ and *CB17 SCID-beige* ^33^ mice, or HCC1954 cells were injected into different location (i.e mammary fat pad) in the host mice^31,33^. In any event, because the efficacy of Enza in inhibiting HCC1954 cell growth *in vitro* and *in vivo*, our data suggest that Enza may be used to overcome trastuzumab resistance.

Our result also reveals that AR antagonist Enzalutamide has efficacy in reducing HER2 + tumor growth in preclinical models. Interestingly, in these preclinical models Enzalutamide as a single agent is just as effective as trastuzumab at inhibiting the growth of HER2 + breast cancer *in vitro* (Fig. 1d) and *vivo* (Fig. 5). Enzalutamide was approved by the FDA to treat castration-resistant prostate cancer in 2012. Considering the promising results of the multiple ongoing clinical trials of Enzalutamide in TNBC and ongoing trial in ER + BC, targeting AR using Enzalutamide could be a useful second line therapy in a subset of HER2 + breast cancer that fail to respond to trastuzumab or develop trastuzumab resistance.

## Materials and Methods

### Cell Culture and Reagents

293T17 cells and breast cancer cell line SKBR3 cells were cultured in Dulbecco’s modified Eagle’s medium (DMEM) (Hyclone) supplemented with 5% fetal bovine serum (FBS) (Sigma), 1 mM sodium pyruvate, 100 units/ml penicillin and 100 μg/ml streptomycin (Hyclone). Breast cancer cell line HCC1954 cells were grown in RPMI-1640 medium (Hyclone) supplemented with 5% FBS, 100 units/ml penicillin and 100 μg/ml streptomycin. All cell lines were cultured in 37 °C humidified tissue incubator with 5% CO2 and 95% air. Enzalutamide was purchased from Selleck and dissolved in DMSO. Trastuzumab (Herceptin) was purchased from Roche.

### Plasmids, lentivirus production, and infection of HCC1954 cells

Lentiviral vector PLKO.1 expressing control-shRNA and two different AR-shRNAs (1 and 2), and packaging plasmids pHR8.9deltaR and CMV-VSVG were purchased from Sigma. 293T17 cells plated in 6-cm dish at 2.5 × 10^6^ cells 24 hours before were cotransfected with 1.88 μg pHR8.9deltaR, 1.25 μg CMV-VSVG, 2.5 μg pLKO1 shRNA using 16.8 μg PEI. Virus supernatants were harvested between 48–72 hours post-transfection, filtered through 0.45 μm low protein-binding sterile filter, and used to infect HCC1954 and SKBR3 cells in the presence of 8 μg of polybrene (Sigma). Pools of HCC1954 and SKBR3 cells expressing shRNAs were selected in the presence of 1.5 μg/ml puromycin (TaKaRa) for 3 days before using for further experiments. Sequences for AR shRNAs are:

ShRNA1: 5′-GAGCGTGGACTTTCCGGAAAT-3′;

ShRNA2: 5′-CACCAATGTCAACTCCAGGAT-3′.

### Colony formation assay

Cells were seeded in 24 well plates at 5000/well in quadruplicate and treated with vehicle control, 20 μM enzalutamide, 20 μg/ml trastuzumab, and combined enzalutamide with trastuzumab. Fresh medium containing the drugs were replaced every two days. Six days later, cells were fixed and stained with 0.5% crystal violet for 20 minutes at room temperature. Crystal violet dye was extracted with 10% acetic acid and quantitated at OD 540 nm using Varioskan flash plate reader (Thermo scientific).

### Western blot analysis

Breast cancer cell lines were lysed in 1X SDS sample (62.5 mM Tris-HCl pH 6.8,2% SDS, 0.002% Bromophenol Blue, 50 mM DTT, 10% glycerol). Tumors from xenografts were homogenized in modified RIPA lysis buffer [1% Triton X-100, 50 mM Tris-HCl, pH 7.4, 1 mM EDTA, 150 mM NaCl, 0.25%Na-deoxycholate, 0.05%SDS, 10 mM NaF, 1 mM sodium vanadate, 1 mM phenylmethylsulfonyl fluoride and protease inhibitor cocktail (sigma)] using Brinkmann Polytron PT2100. Tumor lysates were clarified by centrifugation (>15 K rpm) at 4 °C for 20 minutes. Protein concentrations in clarified lysates were quantified using BCA Protein Assay Kit (Beyotime Institute of Biotechnology) according to manufacturer’s protocol. Equal amounts of cell lysateswere resolved by 8% SDS-PAGE and transferred to Immobilon-P membrane (Millipore Inc.), immunoblotted using the appropriate primary and the HRP-conjugated secondary antibodies (Beyotime Institute of Biotechnology), and developed using enhanced chemiluminescence (ECL) reagent (Thermo Scientific). ECL signals were captured by the ChemiDoc MP imaging system (Bio-Rad) and were analyzed using the Image Lab 4.0 software (Bio-Rad). Alpha -Tubulin (Abcam) was used as the loading control. Monoclonal antibody (mAb) against ErbB2 was purchased from Santa Cruz Biotechnology. Antibodies recognizing phospho-HER2 (Tyr1221/1222) (P-HER2), HER3, phospho-HER3(Tyr1197) (P-HER3), cleaved Caspase-3(9G10), phospho-Akt (P-Akt) (Ser473) and phospho-P44/42 MAPK (Erk1/2) (Thr202/Tyr204) (P-Erk) were from Cell Signaling Technology. Antibodies recognizing AR (06–680) was from Merck Millipore.

### HCC1954 Xenograft model

Protocols for the animal studies were approved by the Institutional Animal Care and Use Committee of Wenzhou Medical University. All animal experiments were performed in accordance with the relevant guidelines and regulations. BALB/c female nude mice (6 to 8 week old) purchased from Shanghai SLAC laboratory animal center were used for the  *in vivo* experiments. HCC1954 cells (2 × 10^6^/100 μl) were mixed with 100 ul growth factor reduced Matrigel (BD Bioscience) and injected subcutaneously at the flanks of the mice. Tumor growth was measured by caliper measurement every 3 days. The modified ellipsoid formula was used to calculate the tumor volume: (width)^2^ × (length)/2. Once all the tumors reached the tumor volume of 300–500 mm^3^ (~15 days after injection), four group of mice were randomized and treated with the following four regiments: 1) vehicle control: 2.7% DMSO + 30% PEG300 plus PBS; 2) 25 mg Enzalutamide/kg plus PBS; 3) 2.7% DMSO + 30% PEG300 plus 10 mg trastuzumab/kg; 4) 25 mg Enzalutamide/kg plus 10  mg trastuzumab/kg. Enzalutamide was dissolved in 2.7% DMSO, formulated in 30% PEG300 and administrated daily to mice by oral gavage. Trastuzumab was dissolved in PBS and injected intraperitoneally twice a week. Twenty one days after treatments, the animals were sacrificed after the last measurement of tumor size. Tumor growth was presented as fold change in tumor volume, which was calculated by dividing the tumor volume on a given day with the tumor volume on day 3 after injection of cells into mouse flank. The tumor tissues were dissected, fixed in 10% formalin for 24 hours and processed for immunohistochemistry or flash-frozen in liquid nitrogen and stored at −80 °C for western blot analysis later.

### Hematoxylin and Eosin (H&E) Staining and Immunohistochemistry

Sections (5 μm thickness) of the formalin-fixed, paraffin-embedded (FFPE) tumor tissues were deparaffinized in xylene, rehydrated with a series of graded ethanols, then stained in Hematoxylin solution for 15 min and counterstained in eosin solution using the Hematoxylin and Eosin Staining Kit (Solarbio).

For immunohistochemistry, sections were deparaffinized, rehydrated as above, and subjected to heat induced epitope retrieval in 10 mM citrate buffer, pH 6.0 using Supor Pressure Cooker (at 125 °C for 5 min). Slides were immersed in 3% H_2_O_2_ for 8 min at room temperature (RT), then blocked with 10% normal goat serum (Solarbio) diluted in PBS for 20 min at RT, and incubated with Ki-67 mouse mAb (1:1200) (Cell signaling technology) and cleaved caspase 3 rabbit mAb (1:400) (Cell signaling technology) overnight at 4 °C. Normal mouse IgG (Beyotime#A7028) (1:1200) and rabbit IgG (Beyotime#A7016) (1:200) were used as negative controls. For detection of Ki-67, polyperoxidase conjugated anti-mouse/rabbit IgG Fab (ZSGB-BIO) was used followed by incubation with 3, 3′-diaminobenzidine chromogen DAB detection Kit (ZSGB-BIO). For detection of cleaved caspase 3, biotin labeled anti-rabbit secondary antibodies was used followed by incubation with streptavidin-horseradish peroxidase (SABC kit SA1052) (Boster) and 3, 3′-diaminobenzidine chromogen DAB detection Kit. Sections were counterstained with diluted hematoxylin for 2 min before mounted. For quantification of Ki-67 and cleaved caspase 3 IHC, images from ten random representative 400 × fields per mouse tumor were captured by Nikon Eclipse 80i Microscope. Ki-67 and cleaved caspase 3 staining positive cells in each field were counted and the average of positive staining cells per field was presented. The IHC data shown was representative from three independent experiments.

### Statistical Analysis

Statistical analyses were performed using SPSS, version 19.0. Quantitative data from at least three independent experiments were expressed as mean ± S.E.M. Student’s t-test was used to compare data between two groups. One-way ANOVA with Bonferroni’s multiple comparison test correction was used to analyze data among multiple groups. All statistical tests were two-sided and P < 0.05 was considered statistically significant.

## Electronic supplementary material

Supplementary Information
