# Supplementary material for: Targeting Androgen Receptor in Treating HER2 Positive Breast Cancer
Source: Sci Rep. 2017 Nov 6;7:14584. doi: 10.1038/s41598-017-14607-2 (PMC5674043; doi:10.1038/s41598-017-14607-2)

**Targeting Androgen Receptor in Treating HER2 Positive Breast cancer**

Licai He^1#^, Zhuanyun Du^1#^, Xusheng Xiong^1^, Hua Ma^1^, Zhenfeng Zhu^1^, Hongwei Gao^1^, Jiawei Cao^1^, Tong Li^1^, Hongzhi Li^1^, Kaiyan Yang^2^, Guorong Chen^2^, Jennifer K. Richer^3^*, Haihua Gu^1,3^*

^1^Key Laboratory of Laboratory Medicine, Ministry of Education, School of Laboratory Medicine and Life Science, Wenzhou Medical University, Wenzhou 325035, China; ^2^Department of Pathology, The First Affiliated Hospital of Wenzhou Medical University, Wenzhou Medical University, Wenzhou 325035, China; ^3^Department of Pathology, University of Colorado Anschutz Medical Campus, Aurora, Colorado 80045, USA

^#^ These authors contribute equally to this work.

*Correspondence: Dr. Haihua Gu, School of Laboratory Medicine and Life Science, Wenzhou Medical University, Chashan Higher Education Park, Wenzhou, Zhejiang 325035, China, Email address: haihuagu@wmu.edu.cn

Dr. Jennifer Richer, Department of Pathology, University of Colorado Anschutz Medical Campus, Aurora, Colorado 80045, USA, Email address: Jennifer.Richer@ucdenver.edu.

Running title: AR in HER2+ breast cancer

Key words: Androgen receptor, HER2 phosphorylation, HER2+ breast cancer

**Supplementary Information**


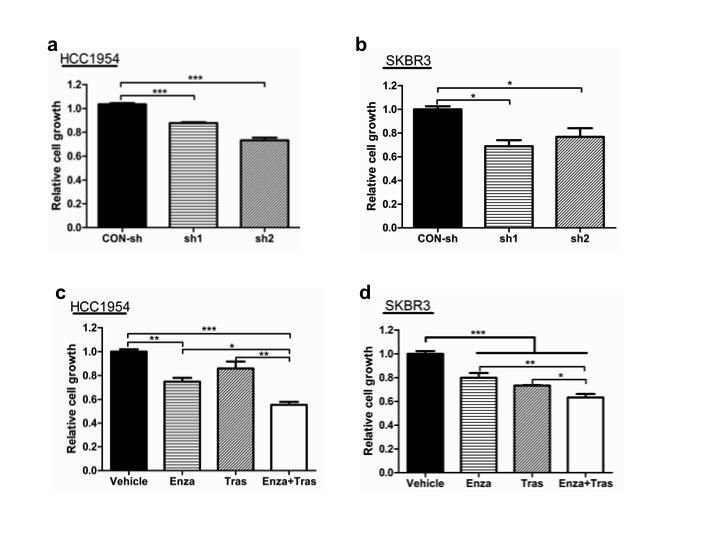
Supplementary Figure S1. Inhibition of AR impairs the growth of HER2 breast cancer cells. (a,b) Knockdown of AR reduced the growth of HCC1954 (a) and SKBR3 (b) cells. Cells expressing control-shRNA (Con-sh), AR-shRNA1 (sh1), AR-shRNA2 (sh2) were plated in 96-well for 6 days before adding Cell Counting Kit-8 (CCK-8) reagent (Dojindo Molecular Technologies, Inc, Japan) to measure the relative amount of viable cells (See manufacture instruction). (c, d) AR antagonist, enzalutamide (Enza), together with trastuzumab (Tras) inhibited the growth of HCC1954 (c) and SKBR3 (d) cells. Cells plated in 96-well were treated with Vehicle, 20 μM Enza, 20 μg/ml Tras, and 20 μM Enza+20 μg/ml Tras for 6 days before adding CCK8 reagent to measure viable cells. The relative number of viable cells for Con-sh cells or cells treated with vehicle was set as 1. Data shown is the representative from three independent experiments. *p<0.05, **p<0.01, and ***p<0.001, one-way ANOVA.

Supplementary Figure S2. Full-length blots for Figures 1a and 1b, bottom panel


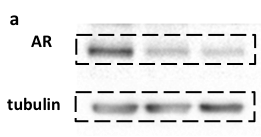

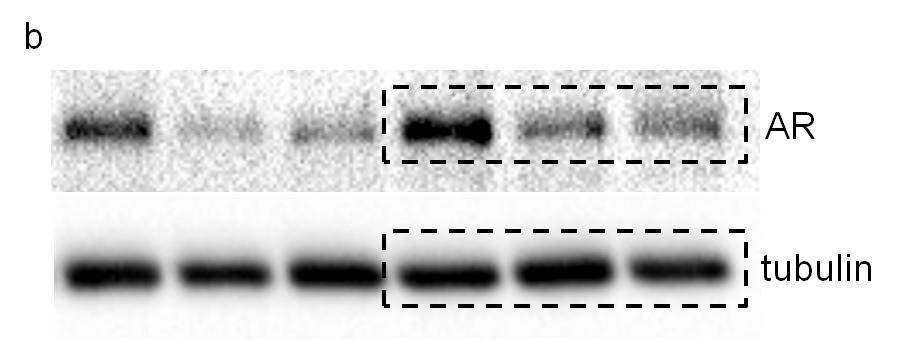


Supplementary Figure S3. Full-length blots for Figures 2a


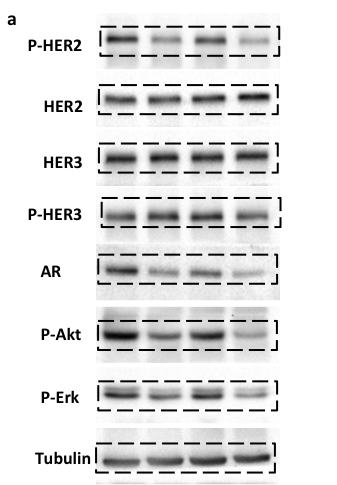


Supplementary Figure S4. Full-length blots for Figures 3a


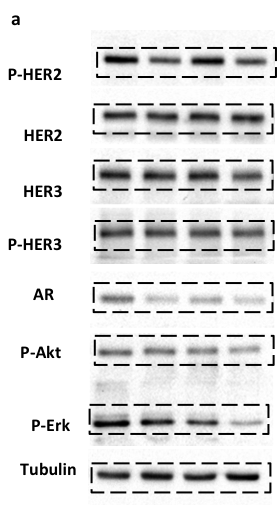


Supplementary Figure S5. Full-length blots for Figures 4a


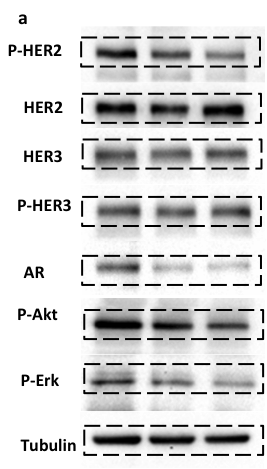


Supplementary Figure S6. Full-length blots for Figures 7a


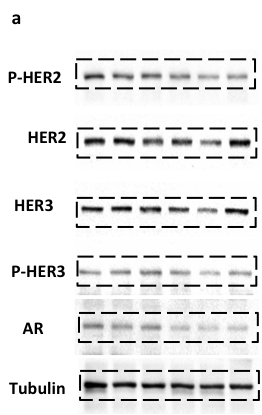

Supplement: Supplementary file 1 — Supplementary Information [file 41598_2017_14607_MOESM1_ESM.doc]
